# Effectiveness of Mulligan’s Two-Leg Rotation Versus Muscle Energy Technique in Subjects With Hamstring Tightness

**DOI:** 10.7759/cureus.28890

**Published:** 2022-09-07

**Authors:** Pratik R Jaiswal, Irshad Qureshi, Pratik A Phansopkar

**Affiliations:** 1 Physiotherapy, Ravi Nair Physiotherapy College, Datta Meghe Institute of Medical Sciences, Wardha, IND

**Keywords:** modified oswestry disability questionnaire, lumbar rom, active knee extension, hamstring tightness, muscle energy technique, mulligan’s two leg rotation

## Abstract

Background

Hamstring stiffness has been growing more common, but is often neglected. The hamstring muscle complex is the key and most commonly involved muscle group for stiffness, and the younger generation is particularly susceptible. The tightness causes a limited range of motion and associated consequences. The purpose of this study is to look for the efficacy of Mulligan’s two-leg rotation (TLR) and muscle energy technique (MET).

Methods

To evaluate TLR against MET, an intervention with a duration of six days per week was planned. Modified Oswestry disability questionnaire (MODQ), active knee extension (AKE), lumbar range of motion (LROM), and numerical pain rating scale (NPRS) were used as outcome measures. The duration of the study was six months. It is a pre and post-interventional type of study.

Result

Statistical analysis was done by using descriptive and inferential statistics using Student’s paired and unpaired t-tests. The Statistical Package for Social Sciences (SPSS) version 27 (IBM Corp., Armonk, NY, USA) was used. A p-value <0.05 was considered significant. Both the treatment protocol were beneficial for the patients but TLR yields a more significant reduction in tightness and pain than MET.

Conclusion

The results after the data analysis show that TLR should be utilized for individuals with hamstring tightness because it exhibited a significant reduction in tightness and pain when compared to MET.

## Introduction

The hamstring muscle is likely the most commonly affected muscle group due to excessive stress [1]. The hamstring, which resists knee extension, is constituted of semitendinosus, semimembranosus, and biceps femoris muscles [2]. Sitting for long periods may result in reduced hamstring mobility [3]. The lumbopelvic rhythm is affected by tight hamstrings. It indirectly affects the stability of the sacroiliac joint. Flexibility is key to fitness and is necessary for both sports and everyday activities [4]. Patellar tendinopathy and patellofemoral soreness, as well as hamstring injury and muscle injury symptoms following eccentric activity, are all connected to decreased hamstring mobility [5]. Due to inactivity and irregular exercise, hamstring tightness is common and frequently occurs in people with non-specific low back pain (LBP) [6]. Mulligan's methods were proven to improve hamstring mobility. It's particularly useful for people who have a substantial symmetrical deficit in straight leg raise (SLR) [7]. Two-leg rotation (TLR) is a simple method that could be used on anyone who has tight hamstrings, low back pain, and restricted or uncomfortable SLR [8]. Muscle energy technique (MET) is an osteopath-developed manual method now employed in a variety of physical treatment specialties, including physiotherapy, massage therapy, and athletic training centers [9]. Muscle strengthening, elongating a shorter or constricted muscle, serving as lymphatic or a venous pump to assist in blood or fluid draining, and widening the range of motion (ROM) of a limited joint are all stated benefits of MET [10]. The MET has been demonstrated to have a greater benefit than static stretching since it lowers pain and discomfort and generally causes further changes in the target tissue, either acutely or long-term [11]. The improved ROM after the contract-relax workout regimen could be due to an improvement in stretch tolerance [12]. When paired with neuromuscular re-education and resistance training, MET is demonstrated to be helpful in lowering lumbopelvic pain as a single intervention and in lowering disability in acute LBP [13].

## Materials and methods

**Figure 1:**
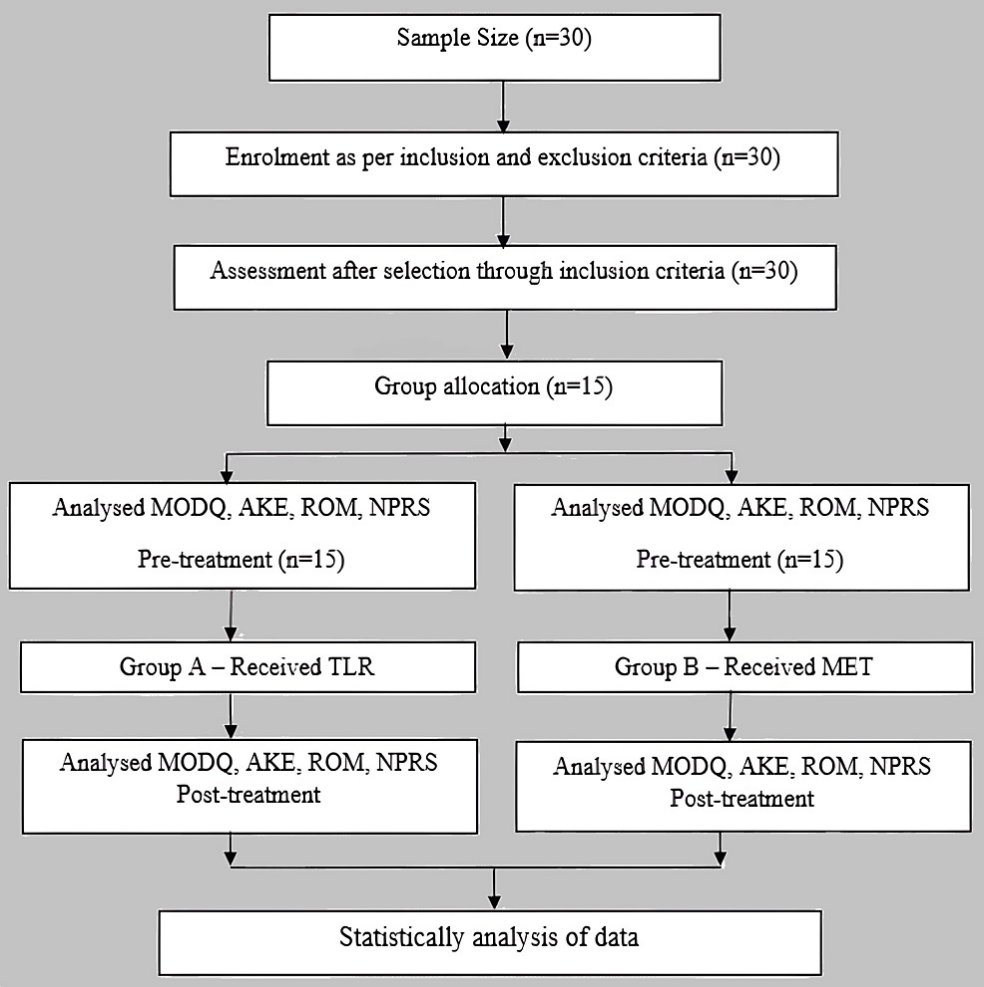
Flowchart of the study procedure

The investigation was carried out in the musculoskeletal outpatient department (OPD) of Acharya Vinoba Bhave Rural Hospital, Wardha, Maharashtra, with approval from the institutional ethics committee of Datta Meghe Institute of Medical Sciences (Deemed to be University) (approval no.: DMIMS[DU]/IEC/2021/378). The subjects were chosen from the orthopedic physiotherapy OPD of this hospital. The participants (n=30) were well informed about the research and provided informed consent. Simple random sampling was done using the sequentially numbered, opaque, sealed envelope (SNOSE) method. Pre and post-interventional assessments were done using the modified Oswestry disability questionnaire (MODQ), active knee extension (AKE), lumbar range of motion (LROM), and numerical pain rating scale (NPRS). Readings were recorded and intervention was given for six weeks. The Mann-Whitney U test was not used. Inclusion criteria included the following: age group 18 to 40 (both male and female), nonathletic persons, AKE greater than 15 degrees, and non-pathological low back pain. Exclusion criteria included pathological LBP, LBP due to traumatic history, osteoarthritis of hip or knee, and loss of function in lower limbs. No participant in the study had any other disease such as diabetes, asthma, or any neurological problems. Thirty participants were selected for the study. They were split into two groups of 15. Group A received TLR, and Group B received MET. A flowchart of the study procedure is shown in Figure 1.

Outcome measures

Modified Oswestry Disability Questionnaire

This questionnaire includes 10 questions that cover various functions. Each component receives a score between 0 and 5, with higher numbers signifying more disability. A percentage is calculated by multiplying the overall score by two [14].

Active Knee Extension & Lumbar Range of Motion

**Figure 2:**
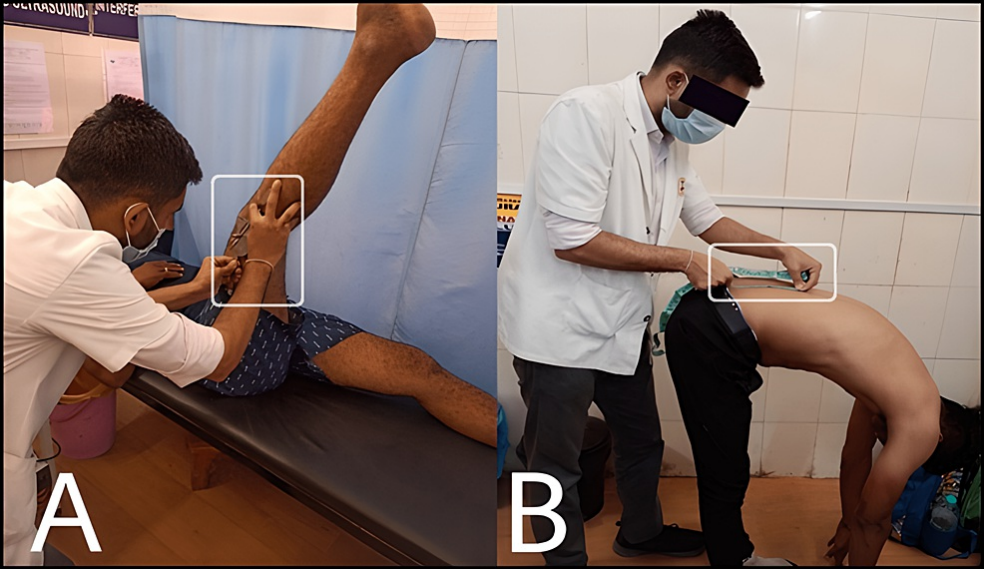
The AKE test using the goniometer (A) and LROM evaluated with a measuring tape (B) AKE: Active knee extension, LROM: Lumbar range of motion

The subject lays on a couch and is then asked to flex the hip to 90^0^. The subject then voluntarily extends the same knee as far as possible while keeping the opposite leg stable on the couch. When the movement is completed, the angle measured by the goniometer is noted as the extension angle (Figure 2). Meanwhile, the LROM is evaluated utilizing a conventional range of measurement methodology by applying a modified Schober's approach using the inch tape (Figure 2).

Numerical Pain Rating Scale

A horizontal line was created on a piece of paper with numerals 0 to 10 where 0 represents no pain and 10 represents the worst acceptable agony, and respondents pointed to a numeral according to the extent of their discomfort and their current level of pain [15].

Intervention

Mulligan's Two-Leg Rotation

The subjects of Group A were made to lie in a supine position. The therapist then gauged the limited hamstring mobility on either side. Legs were flexed on both sides while keeping the shoulders on the treatment table, shifting the subject’s limbs towards the side of decreased mobility. The therapist administered back pressure for 30 seconds after the subject had achieved his or her limit before lowering the legs to the treatment table. This was repeated four times in total resting for one minute between each stretch [6].

Muscle Energy Technique

The subjects of Group B were supine with the treatment side's lower leg placed over the therapist's shoulder. After extending the knee to its first barrier point, moderate (approximately 75% of maximal) isometric contraction of the hamstring muscle was applied for five to eight seconds. After three seconds of relaxation, the technique was repeated three times (a total of four contractions) for five successive days [16].

**Figure 3:**
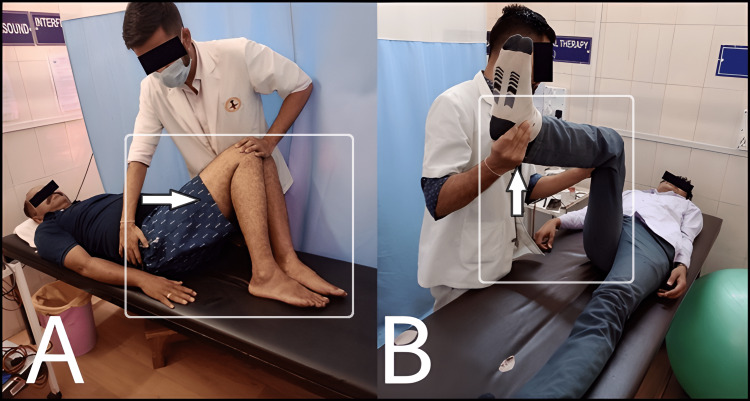
Interventions for hamstring mobility A: Mulligan's two-leg rotation, B: Muscle energy technique

Both intervention techniques are shown in Figure 3.

## Results

The statistical analyses were carried out by utilizing descriptive statistics and inferential statistics, as well as Student's paired and unpaired t-tests. The analysis was done via Statistical Package for Social Sciences (SPSS) version 27 (IBM Corp., Armonk, NY, USA) SPSS 27.0, and a threshold of significance of p<0.05 was evaluated. The results of groups A and B were contrasted to discern which treatment reduced hamstring tightness and improved hamstring flexibility, increased lumbar range of motion, and reduced pain. A paired t-test was performed for comparing pre- and post-scores between groups A and B. The values for the post-mean differences among groups A and B were compared using an unpaired t-test. The comparison of the mean age in years in the two groups is displayed in Table 1. The study participants ranged from 18 to 40 years. The mean age for group A was 31.60 and 27.06 for group B.

**Table 1:** Participant characteristics

Group	N	Mean	Standard Deviation	Standard Error Mean	t-value
Group A	15	31.60	5.51	1.42	2.16 P=0.039,S
Group B	15	27.06	5.94	1.53	

**Figure 4:**
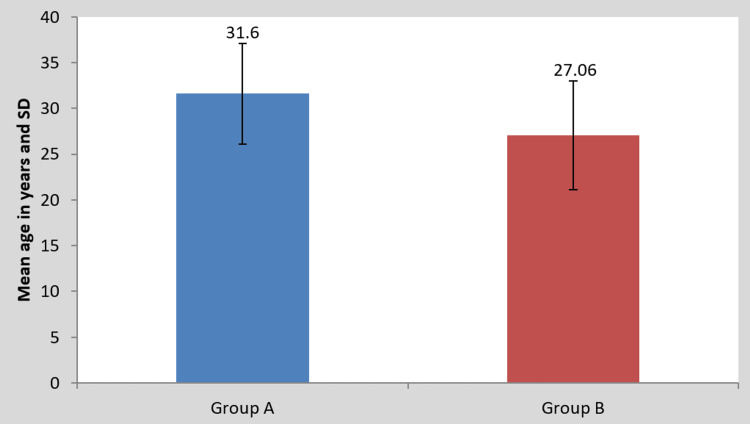
Comparison of the mean age in years in groups A and B SD: Standard deviation

**Figure 5:**
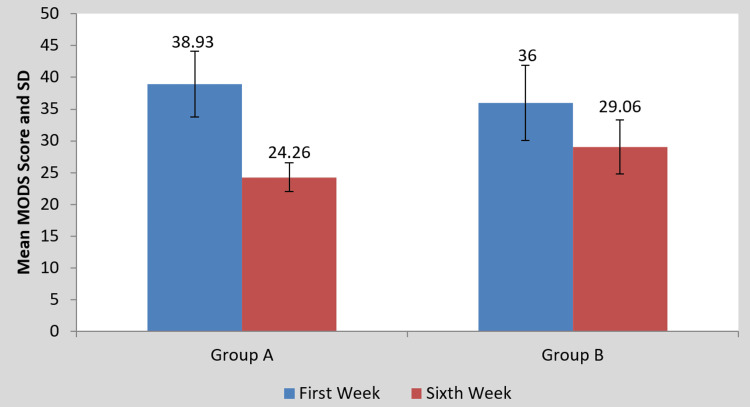
Comparison of MODQ in groups A and B at pre- and post-treatment. MODQ: Modified Oswestry disability questionnaire

**Figure 6:**
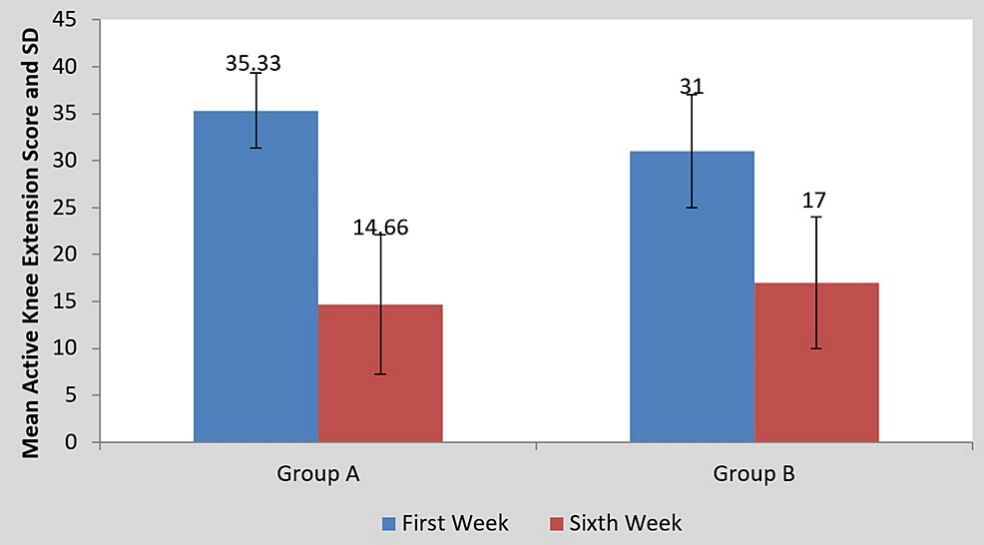
Comparison of AKE scores in the two groups at pre- and post-treatment SD: Standard deviation, AKE: Active knee extension

**Figure 7:**
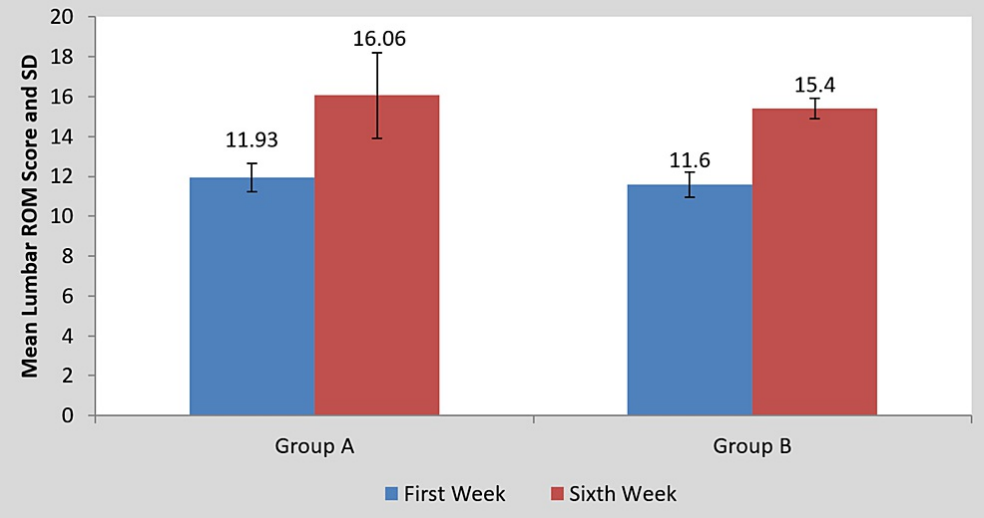
Comparison of Lumbar ROM scores in the two groups at pre- and post-treatment ROM: Range of motion, SD: Standard deviation

**Figure 8:**
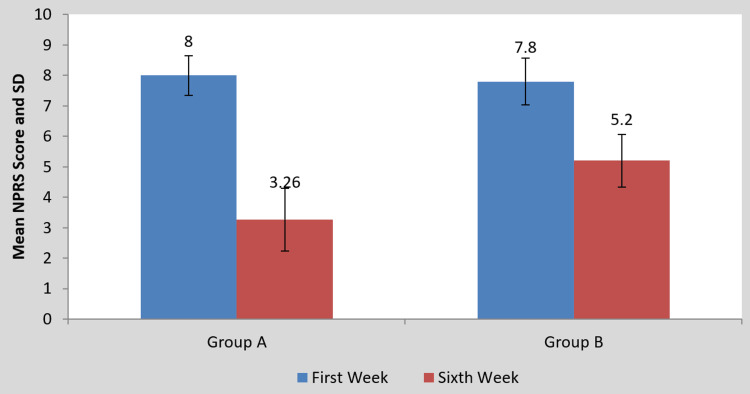
Comparison of NPRS scores in the two groups at pre- and post-treatment NPRS: Numerical pain rating scale

**Figure 9:**
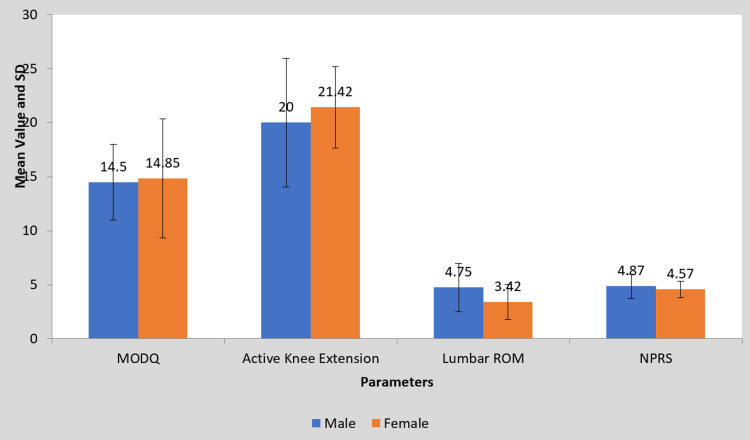
Gender-wise comparison of the mean difference in MODQ, AKE, LROM, and NPRS (group A) MODQ: Modified Oswestry disability questionnaire, AKE: Active knee extension, LROM: Lumbar range of motion, NPRS: Numerical pain rating scale

**Figure 10:**
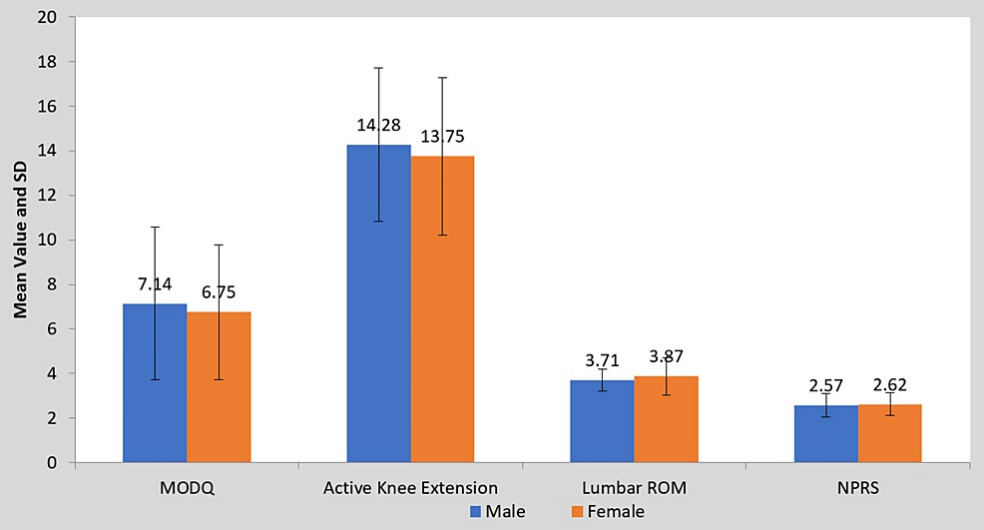
Gender-wise comparison of mean difference in MODQ, AKE, LROM, and NPRS (Group B) MODQ: Modified Oswestry disability questionnaire, AKE: Active knee extension, LROM: Lumbar range of motion, NPRS: Numerical pain rating scale

Statistics illustrating the impact of the intervention on the scores of the MODQ, AKE, LROM, and NPRS are presented in Table 2 and Figures 4-10, respectively. Table 2 shows a statistical analysis of the variables that were measured as well as the significance of the comparison between the pre- and post-intervention results for the groups. Following the intervention compared to before the intervention, scores on outcomes measures significantly decreased in group A than in group B (p<0.0001). However, when the mean difference among the two groups was examined post-intervention, the scores of the MODQ, AKE, LROM, and NPRS in the participants with TLR were greatly reduced. The TLR outperformed MET (p<0.0001), demonstrating a significant difference between the two.

**Table 2:** Mean MODQ, AKE, LROM, NPRS value pre- and post-treatment of groups A and B and between groups A and B. MODQ: Modified Oswestry disability questionnaire, AKE: Active knee extension, LROM: Lumbar range of motion, NPRS: Numerical pain rating scale, SD: Standard deviation.

Outcome Measure	Group A		P-value	Group B		P-value	Mean difference (X±SD)		P-value
	Pre-treatment	Post-treatment		Pre-treatment	Post-treatment		Group A	Group B	
MODQ	38.93±5.17	24.26±2.25	0.0001	36±5.95	29.06±4.26	0.0001	14.66±4.38	6.93±3.10	0.0001
AKE (in degrees)	35.33±3.99	14.66±7.43	0.0001	31±6.03	17±7.02	0.0001	20.66±4.95	14±3.38	0.0001
LROM (in cm)	11.93±0.70	16.06±2.15	0.0001	11.60±0.63	15.40±0.50	0.0001	4.13±2.03	3.80±0.67	0.0001
NPRS	8±0.65	3.26±1.03	0.0001	7.80±0.77	5.20±0.86	0.0001	4.73±0.96	2.60±0.50	0.0001

## Discussion

This investigation compared the efficiency of TLR and MET for tightness in the hamstring of 30 participants. This survey shows that both strategies were shown to be helpful. If we consider gender, in group A females outperformed males in MODQ and AKE while in group B males outperformed females in MODQ and AKE. However, those who received TLR had better recovery in knee range of motion (ROM) and lower NPRS scores, whereas hamstring muscle tension was observed to be lowered more among TLR receivers. The MODQ, AKE, NPRS, and LROM were used as outcome measures in this study. The study's findings demonstrated the importance of TLR in comparison with MET.

Hamstring stiffness is yet another musculoskeletal issue that has arisen as a result of the shift in lifestyle. The population's adaptation to physical activities, particularly the musculoskeletal system, is at risk of degeneration and functional loss due to current practices [17]. Koli et al. in a cross-sectional analysis of the incidence and severity of hamstring tightness in college students found that the incidence of hamstring tightness is quite higher in college students aged 18 to 25. To avoid musculoskeletal problems in the lower extremity it is vital to be aware of hamstring stretching [5]. Various manual treatment approaches have indeed been researched for reducing muscular tension [18]. Established by Fred Mitchell Sr. and Jr., MET utilizes a properly controlled posture in a specified direction to cause a muscle to contract against achieved counterpressure [19]. The MET is a type of physical therapy where a patient uses his or her muscle in a specific manner, disregarding the therapist's counterforce [20]. Mulligan's techniques have previously been proven to improve hamstring flexibility [7]. In his clinical research, Phansopkar et al. discovered that TLR increases hamstring flexibility and ROM, and reduces pain and functional impairment in patients with acute non-specific low back pain. The TLR can therefore be used frequently like other Mulligan procedures in clinical settings to increase the flexibility of the hamstrings [6]. The TLR is a comfortable treatment that can help those with strained hamstrings, lower back agony, and incomplete or uncomfortable SLR. It is especially beneficial for individuals facing symmetrical problems with SLR [8]. The muscles are steadily extended to tolerability in bent leg raise (BLR) and TLR stretching, and the placement held is with the musculature at its greatest bearable extent [21].

The MET-enhanced hamstring flexibility is more than just dynamic stretches. The process of MET for elevating muscle length includes both mechanical factors (like plastic and viscoelastic changes in the connective tissue elements of the muscle) as well as neurophysiological (including changes in stretching tolerances). The efficiency of MET has been accredited to inhibiting Golgi tendon reactions [22]. Thomas et al. in their review report that MET is beneficial in relieving subjective pain, impairment, and joint ROM in both symptomatic and asymptomatic individuals, and there is additional evidence that MET is a fruitful treatment for acute low back aching and improves associated disability indexes [23]. The outcome of MET over the elasticity of hamstring muscle in national football troupes revealed that MET helps in improving hamstring lengthening, which helped them avoid injuries and achieve better flexibility [24].

There are several external standard techniques for determining changes in hamstring length. Active and passive SLR, AKE test, and the sit & reach test are three regularly used tools [25]. The improvement in the SLR range in the traction SLR group could be attributed to the reality that different receptors limit lower limb alpha motor neuron firing throughout the traction SLR stretch. During limb traction, Golgi tendon organs surrounding the knee, hip, and spine likely trigger numerous segmental reflex pathways. Similarly, through large-amplitude stretching motions such as SLR, Golgi tendon organs are engaged. By reducing the afferent activities of type II muscle spindles or reducing motor neuron excitation through 1-b fibers, this nervous system processing may decrease the action of the muscles becoming extended throughout SLR. As a result, improvements in SLR ranges could be directly connected to hamstring muscle restriction instead of alterations in stretch tolerance [26].

The limitations of the study are the sample size, and only healthy individuals were recruited for the study. Large sample sizes should be the emphasis of future research, and the same study can also be conducted in another age range.

## Conclusions

A distinctive worry in today's lifestyle, notably in sedentary ones is stiffness or loss of flexibility across the body. The most typical cause is increased inactivity in people, which is commonly brought on by hamstring tightness. The difficulties will be solved more quickly if the tightness is relaxed. In this study, we examined two commonly used approaches to see which was more efficient. With an equivalent number of sessions for both groups, our research attempted to compare the efficiency of both the TLR and MET techniques. According to the findings, TLR should be utilized for individuals having hamstring tightness because it exhibited a significant reduction in tightness and pain when compared to MET. However, further studies need to be conducted but with a variety in age, larger population, and different outcomes.

## References

[1] Kalanekar TA, Koley S (2020). A comparative study of Mulligan's bent leg raise versus muscle energy technique in asymptomatic individuals with hamstring tightness. EAS J Orthop Physiother.

[2] Kimura A (2021). The effects of hamstring stretching on leg rotation during knee extension. J Phys Ther Sci.

[3] Sojitra N, Shukla Y (2020). A study to compare immediate effect of suboccipital muscle inhibition technique and muscle energy technique on hamstring flexibility in healthy collegiate subjects - an interventional study. Indian J Physiother Occup Ther.

[4] Rabia K, Nasir RH, Hassan D (2019). Immediate effect of muscle energy technique in comparison with passive stretching on hamstring flexibility of healthy individuals: a randomized clinical trial. Isra Med J.

[5] Koli BK, Anap DB (2018). Prevalence and severity of hamstring tightness among college student: a cross-sectional study. IJCBR.

[6] Phansopkar PA, Kage V (2014). Efficacy of Mulligan’s two leg rotation and bent leg raise techniques in hamstring flexibility in subjects with acute non-specific low back pain: randomized clinical trial. Int J Physiother Res.

[7] Laxmi V R (2015). Effectiveness of Mulligan’s two-leg rotation and bent leg raise techniques in subjects with acute non specific low back pain in improving hamstrings flexibility. TJPRC: IJPOT.

[8] Taru A, Jeswani K (2018). Study to compare the effectiveness of Mulligan's bent leg raise and two-leg rotation techniques on hamstring flexibility in amateur football players. GJRA.

[9] Choksi P, Tank K: To Study the (2016). Efficacy of muscle energy technique on muscle strength and flexibility in patients with knee osteoarthritis. Indian J Physiother Occup.

[10] Srithren NS, Sundaram SS (2020). The effect of muscle energy technique on flexibility of hamstring muscle in futsal players. Malays J Mov Health Exerc.

[11] Azizi M, Shadmehr A, Malmir K, Qotbi N, Khazaei Z (2021). The immediate effect of muscle energy technique and whole body vibration on hamstring muscle flexibility and stiffness in healthy young females. Muscles, Ligaments Tendons J.

[12] Majeed A, Mansoor SR, Arif AB, Yasin MM, Wasim M, Naeem F (2021). Comparison of static stretching and muscle energy techniques on hamstring tightness in asymptomatic females. FUJRS.

[13] Tikhile PJ, Kulkarni CA, Bele AW (2021). Comparative study of efficacy of cryotherapy and myofascial release technique in calf muscle spasticity in spastic diplegic cerebral palsy children. JMPAS.

[14] Fritz JM, Irrgang JJ (2001). A comparison of a modified Oswestry low back pain disability questionnaire and the Quebec back pain disability scale. Phys Ther.

[15] Childs JD, Piva SR, Fritz JM (2005). Responsiveness of the numeric pain rating scale in patients with low back pain. Spine (Phila Pa 1976).

[16] Talapalli R, Sheth M (2014). Comparison of muscle energy technique and post isometric relaxation on hamstring flexibility in healthy young individuals with hamstring tightness. IJHRS.

[17] Shujat H, Dustgir A, Perveen W, Anwar S, Amin I, Hashmi R (2020). The effectiveness of Mulligan techniques to increase the flexibility of hamstring muscles: a quasi-experimental study. Pak J Med Health Sci.

[18] Joshi TM, Wani SK, Shyam A, Sancheti P (2017). Immediate effects of two different types of muscle energy techniques (MET) on hamstring muscle flexibility in young healthy females: a comparative study. Int J Health Sci Res.

[19] Biswas S, Alagingi NK (2018). Compare the effectiveness of static stretching and muscle energy technique on hamstring tightness among student population. Int J Yoga Physiother.

[20] Tariq K, Shoukat F, Ahmed U (2020). Effectiveness of Mulligan's bent leg raise technique versus muscle energy technique on pain intensity and hamstring flexibility in patients with knee osteoarthritis. Rawal Medical Journal.

[21] Khatri S, Yeole U, Kurle S (2018). Effects of various Mulligan techniques on hamstring muscle imbalance and lumbar spine mobility in marathon runners: a randomized control trial. IJPESH.

[22] Zahra R-S, Mohamad RS, Fatemeh B (2021). Comparison of the effects of static stretching and muscle energy technique on hamstring flexibility, pain, and function in athletes with patellofemoral pain. J Adv Pharm Edu Res.

[23] Thomas E, Cavallaro AR, Mani D, Bianco A, Palma A (2019). The efficacy of muscle energy techniques in symptomatic and asymptomatic subjects: a systematic review. Chiropr Man Therap.

[24] Ruparelia H, Patel S (2019). Immediate effect of muscle energy technique (MET) and positional release therapy (PRT) on SLR90⁰-90⁰, ankle dorsiflexion range and Y-balance test—an experimental study. Int J Health Sci Res.

[25] Hopper D, Conneely M, Chromiak F, Canini E, Berggren J, Briffa K (2005). Evaluation of the effect of two massage techniques on hamstring muscle length in competitive female hockey players. Phys Ther Sport.

[26] Kapadia PD, Meshram VK (2019). A comparative study on immediate effects of traction straight leg and bent leg raise on hamstring muscle flexibility in normal individuals. Int J Appl Res.

